# PACE Continuous Innovation Indicators—a novel tool to measure progress in cancer treatments

**DOI:** 10.3332/ecancer.2015.498

**Published:** 2015-01-07

**Authors:** Silvia Paddock, Lauren Brum, Kathleen Sorrow, Samuel Thomas, Susan Spence, Catharina Maulbecker-Armstrong, Clifford Goodman, Michael Peake, Gordon McVie, Gary Geipel, Rose Li

**Affiliations:** 1 Rose Li and Associates, Inc., Bethesda, Maryland 20817, USA; 2 Prevention and Health Promotion, State of Hessen, 65187 Wiesbaden, Germany; 3 Center for Comparative Effectiveness Research, The Lewin Group, Falls Church, Virginia 22042, USA; 4 University of Leicester, LE1 7RH, United Kingdom; National Lung Cancer Audit, Clinical Effectiveness and Evaluation Unit, Royal College of Physicians, London NW1 4LE, United Kingdom; National Cancer Intelligence Network, London SE1 8UG, United Kingdom; 5 European Institute of Oncology, Milan 20146, Italy; University of Milan, Italy; University of Glasgow, G12 8QQ, United Kingdom; University of Wales, Cardiff, South Glam CF10 3NS, United Kingdom; Founding Editor of *ecancer.org*; 6 Lilly Oncology, Indianapolis, Indiana 46285, USA

**Keywords:** cancer, innovation, value, indicators, progress

## Abstract

Concerns about rising health care costs and the often incremental nature of improvements in health outcomes continue to fuel intense debates about ‘progress’ and ‘value’ in cancer research. In times of tightening fiscal constraints, it is increasingly important for patients and their representatives to define what constitutes ’value’ to them. It is clear that diverse stakeholders have different priorities. Harmonisation of values may be neither possible nor desirable. Stakeholders lack tools to visualise or otherwise express these differences and to track progress in cancer treatments based on variable sets of values.

The Patient Access to Cancer care Excellence (PACE) Continuous Innovation Indicators are novel, scientifically rigorous progress trackers that employ a three-step process to quantify progress in cancer treatments: 1) mine the literature to determine the strength of the evidence supporting each treatment; 2) allow users to weight the analysis according to their priorities and values; and 3) calculate Evidence Scores (E-Scores), a novel measure to track progress, based on the strength of the evidence weighted by the assigned value.

We herein introduce a novel, flexible value model, show how the values from the model can be used to weight the evidence from the scientific literature to obtain E-Scores, and illustrate how assigning different values to new treatments influences the E-Scores.

The Indicators allow users to learn how differing values lead to differing assessments of progress in cancer research and to check whether current incentives for innovation are aligned with their value model. By comparing E-Scores generated by this tool, users are able to visualise the relative pace of innovation across areas of cancer research and how stepwise innovation can contribute to substantial progress against cancer over time. Learning from experience and mapping current unmet needs will help to support a broad audience of stakeholders in their efforts to accelerate and maximise progress against cancer.

## Background

The ongoing ’War on Cancer’ poses a far greater challenge than originally anticipated, largely because solid tumours have been found to be extremely genetically heterogeneous. Experts now estimate that more than 200 distinct types of tumours exist [1]. Additional complexity stems from the ability of these diverse tumours to evolve and develop resistance against treatments, requiring cancer researchers and clinicians to continuously expand and refine their armament [2].

In the meantime, divergent opinions and selective use of indicators continue to enable misunderstandings about advances in cancer research. The public tends to expect progress to occur in great leaps. Once a cure is available (for example in testicular cancer), we tend to forget about the numerous steps it took to achieve that success. Because of space and resource limitations, stakeholders often focus on ‘milestones’ in cancer research, which may augment the flawed view that cancer progress occurs in great leaps. While major advances do occur in irregular, unpredictable intervals, most progress happens more slowly as continuous stepwise improvements accumulate. Educating the public and stakeholders about the true nature and value of stepwise progress against cancer is, therefore, of great relevance [3].

Rising health care costs and the often incremental improvements in health outcomes have fueled intense debates about maximising the ‘value’ of investments in cancer research [4–7]. In times of tightening fiscal constraints, it is increasingly important for patients and their representatives to define what constitutes ‘value’ to them. Different individuals and groups may, for example, weigh risks, benefits, and costs differently. It is clear that different stakeholders representing different interests are unlikely to harmonise their values, which may be determined by the nature of the specific disease, immediate needs and priorities, perceived opportunities for scientific advancement, and competing personal, societal, and economic interests. Indeed, values can change over time even within a homogeneous group of stakeholders. Harmonisation of values, therefore, not only is an unlikely outcome but also may not even be desirable.

Instead of trying to homogenise diverse values, we need a better understanding of existing differences to implement flexible policies and account for real differences in values. Stakeholders lack tools to visualise and otherwise express differences in values, which ultimately leads to different decisions about research and health spending priorities.

## Introducing scientifically rigorous progress trackers

PACE (an initiative of Lilly Oncology, a business unit of Eli Lilly and Company) has responded to these needs by developing objective, scientifically rigorous, and forward-facing indicators that permit tracking and comparing cumulative progress across various cancer types.

A central component of progress in cancer treatments is the knowledge that researchers gain from clinical trials. Every time researchers achieve even a small increase in survival—and often when treatments fail entirely—they gain some understanding of the cancer disease process. Many such small steps ultimately lead to substantial gains. Successful and unsuccessful treatment trials often provide important clues regarding the best next steps. Treatments that we take for granted today often took a long time and many trials to establish.

### Innovation Indicators overview

The Continuous Innovation Indicators allow users to quantify and visualise how stepwise innovation can contribute to more substantial progress against cancer over time. We envision the final online implementation of the Indicators as a simple user interface that generates summary graphs from which a user can access the supporting individual evidence records and additional detailed information (Figure 1).

Evidence Scores (E-Scores) are a novel quantitative measure of cumulative evidence that is further explained below. The user can move up and down between the E-Score summaries and other layers to map the available evidence onto the current Value Matrix, a flexible new platform for weighing the value of treatments based on therapeutic goals. Those interested in the underlying studies can access the exact identifiers of the clinical trials, retrospective studies, systematic reviews, and literature references that comprise the evidence base for a treatment of interest. The first release will include data for 12 anatomical disease sites: breast, colon, endometrium, kidney, liver, lung, pancreas, prostate, skin (melanoma), stomach, rectum, and testes.

Accountability in all steps is essential for the success of this effort because stakeholders will expect to be able to scrutinise the data and the computations that led to the summary scores. The layered design of the Indicators ensures that all stakeholders can access information at the desired level.

### Innovation data management: creating a flexible resource

One factor that has likely slowed progress in the past is the lack of a common framework to assess areas in which research has progressed versus those that have lagged behind. PACE has initiated this project to implement data management procedures that can track progress consistently across cancers. Figure 2 provides an overview of these procedures and resulting outputs.

To convert data from PubMed references and other sources into quantitative measures, we have developed a standardised data acquisition and management methodology with the following main components:

- **Pieces of Evidence:** When reviewing evidence from the literature, one complicating factor is the frequent use of multiple interventions and outcome measures within the same study. Users may have different views of or uses for the findings. We therefore developed a methodology to disaggregate the evidence by splitting each study into distinct Pieces of Evidence. From each study, we derive one or more such pieces, each of which contains one disease state, therapeutic goal, treatment, and outcome. For each outcome, we include only analyses that apply proper correction for multiple testing.
- **Evidence Hierarchy/Quality:** Evidence that a new treatment works usually accumulates over time. To derive a quantitative measure, we set thresholds for evidence of sufficient strength to count and to define milestones for achieving stronger evidence. We further developed a system to assign weights to evidence from different kinds of studies (e.g. meta-analyses, randomised controlled trials (RCTs), observational studies). This can be done at the level of the type of study or for each individual study.
- **Value Matrix:** To measure progress, we need to define ‘success’. Which developments are sufficiently different from existing treatments to be considered ‘novel’? Which are ‘improvements’ of existing treatments? How do we value different treatment goals? We have developed a novel Value Matrix for cancer research, which is further explained below. Analysts working with the system who want to set their priorities differently can easily change the parameters.
- **Relational database with tagged data:** Our audiences will be interested to compare outcomes not only between anatomical sites of the primary tumour, but also between different therapeutic approaches (e.g. targeted therapy versus immunotherapy) or even between individual targeted signalling pathways. The Continuous Innovation Indicators use a relational database design and data-tagging methods to provide this flexibility. This approach allows comparisons across anatomical sites, pathways, treatments, etc. It is an important feature to create forward-looking indicators that can reveal important data stratified by not only disease site but also with emerging biomarkers and therapeutic approaches.

Output formats include detailed plots of the data onto the Value Matrix. We further supply E-Scores to measure progress over time and summary statistics that can be stratified by a large number of tags (e.g. molecular and histological subtypes, early versus late onset, disease stages, or the time it takes different international regulatory agencies to approve treatments).

## Methods

### Continuous Innovation Indicators Pieces of Evidence: a novel unit to measure evidence

**Figure 3.**
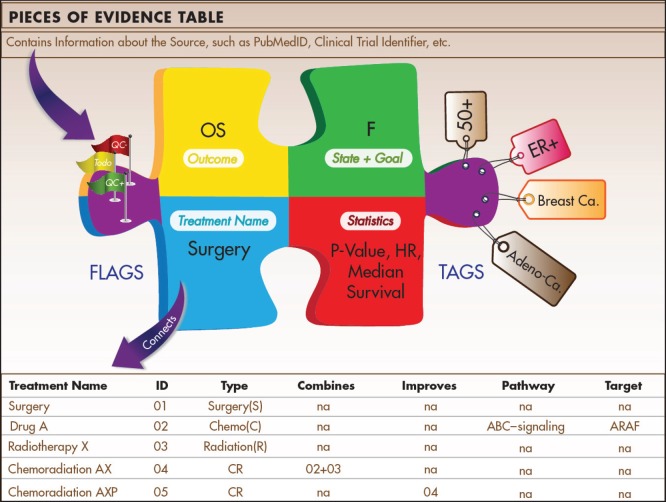
Pieces of Evidence. Pieces of Evidence are the units of analysis for the Indicators. Each piece reports statistics for a specific outcome (e.g. overall survival) of a treatment tested in a specified disease state (e.g. metastatic) with a specific therapeutic goal (e.g. become disease-free). The disease state and treatment goal are classified in an alphabetic system derived from the Value Matrix. Tags contain information on applicable patient subgroups (e.g. histological subtypes, biomarker status, age), and flags contain internal information about quality control procedures. Pieces of Evidence are stored in a data table that is part of a relational database. The relational design allows users to sort and analyse evidence in multiple ways.

The Indicators approach relies initially on disaggregating the evidence. Each publication, clinical trial record, historical reference, or other source of information usually reports multiple analyses of outcomes. These measurements become separate Pieces of Evidence (Figure 3) in our system. Each piece reports the test results for one pre-planned measurement in one context, for example:

- One Piece of Evidence for each treatment arm, for example:
  - Drug A / Drug B versus placebo
  - Drug A versus Drug B, if Drug B is the current standard of care
  - Drug A and Drug B together vs. Drug A and Drug B administered sequentially
- One Piece of Evidence for each time point (e.g. initial results versus long-term follow-up)
- One Piece of Evidence for each patient subgroup (e.g. entire population versus subgroup positive for a molecular marker)
- One Piece of Evidence for each analysis goal (e.g. superiority versus non-inferiority of health outcomes)

Each Piece of Evidence can contain multiple tags that provide additional information about the anatomical site, eligibility criteria, histological subtypes, etc. Additional information about each Piece of Evidence is gathered in a separate data table that is part of a relational database. This ensures that parameters in the Piece of Evidence table are connected to additional tables. The treatment table, for example, contains information about the drug class, the type of surgery or radiotherapy, different approaches of targeted therapies, and eligibility criteria (e.g. ‘postmenopausal’). It further links treatments to other treatments and tracks whether a treatment is considered a new treatment, an improvement of an existing treatment, or a combination of several existing treatments.

This flexible approach allows the user to query the database and retrieve all evidence supporting or discounting a certain type of targeted treatment (e.g. ‘anti-angiogenesis treatment’) in a given context (e.g. advanced colorectal versus gastric cancer). Through the PubMed identifier, each Piece of Evidence is tagged with a date that further allows users to track progress over time and visualise possible lags in innovative developments in one cancer versus another.

For the analysts working on the database and populating it with new Pieces of Evidence, the relational design makes the daunting challenge of reviewing the evidence less cumbersome, because it divides it into manageable units of analysis. The analysis team can further work on the Pieces of Evidence and treatment characterisations in parallel. Quality control procedures for data in each table ensure that only valid information becomes connected to other parts of the database and is included in the E-Score calculations. To facilitate this process, each Piece of Evidence carries multiple flags that can assume yes/no/to-do values to help with the systematic review of all data.

We have incorporated several safeguards to ensure data and analytical integrity. Data review is based on automated inputs and downloads of electronic records through the National Center for Biotechnology Information (NCBI) Ebot tools, which minimises manual typing errors and saves time for analysts. All analysts use a common standard operating procedure to determine which evidence to include. Finally, multiple analysts conduct blind duplicate reviews to ensure consistent scoring.

Appendix 1 explains potential sources of variability, strategies used to maximise consistency, and estimates of how often remediation is needed.

### Continuous Innovation Indicators E-Scores: a novel measure of progress

Identifying the best measure to track progress in cancer research is not trivial. Several seemingly ‘obvious’ candidate measures fail to provide an accurate picture when scrutinised closely. Period survival, for example, is subject to lead-time and length biases [8].

The number of cancer survivors, which is sometimes used as an indicator of success, has increased during the past decades and is projected to continue to increase in the coming years. The definition of ‘survivor’, however, is a person who has been diagnosed with cancer and is still alive [9]. Based on this definition, survivorship is heavily dependent on population’s age distribution and timing of diagnosis. Because the population of most developed countries is ageing, we expect to see more ‘survivors’ simply because of the fact that older age is one of the largest risk factors for most cancers [10]. The number of survivors is further biased by earlier diagnosis of occult tumours and is thus unfit to provide an accurate index of progress against different cancers. It is also difficult to attribute increases in the number of survivors to specific advances in treatment.

**Figure 4.**
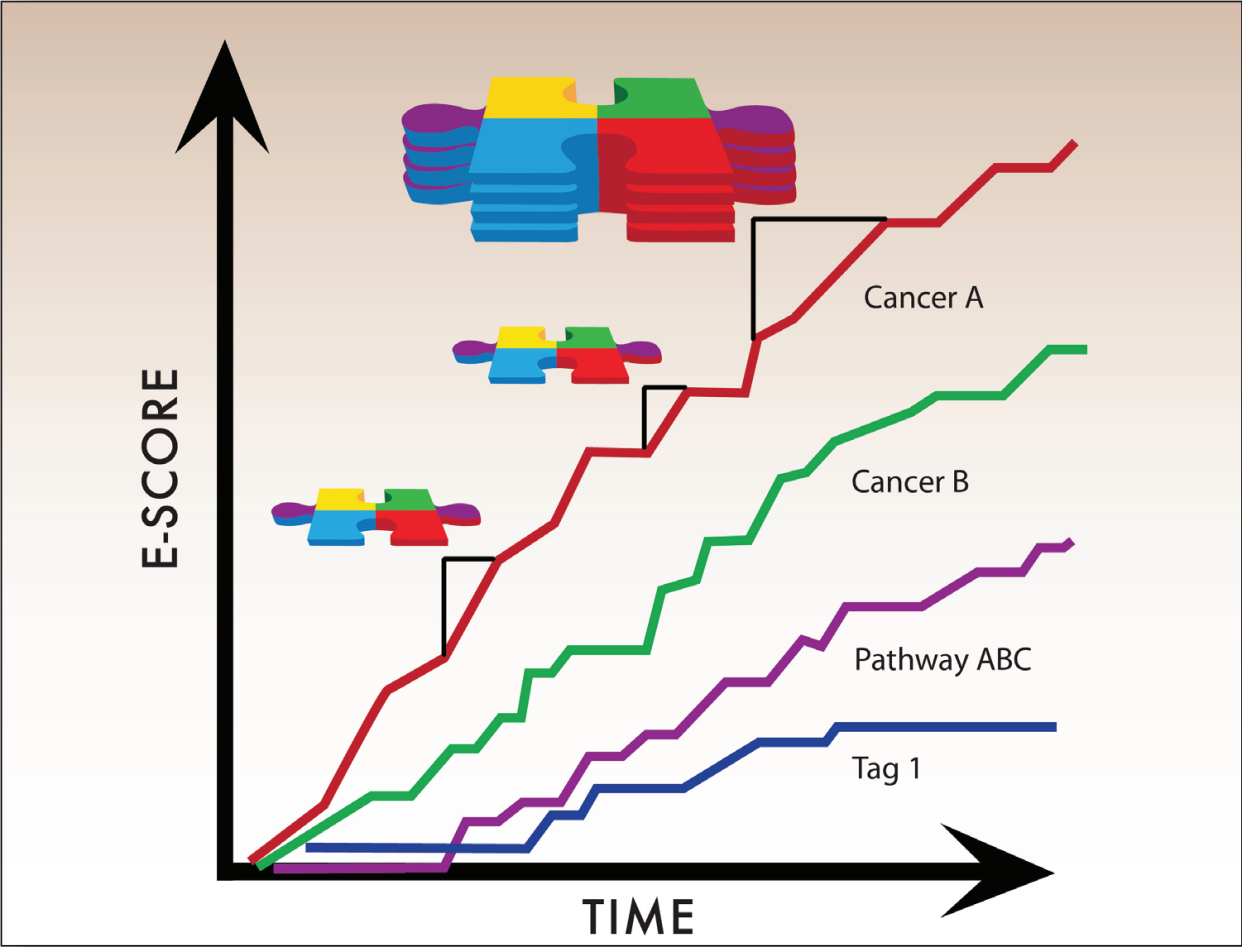
E-Scores are the sum of accumulating Pieces of Evidence over time. E-Scores, therefore, increase with each new Piece of Evidence that meets user-defined criteria. The general mathematical formula for E-Scores is: evidence x value weights x custom weights. The amount by which individual Pieces of Evidence raise the E-Score thus depends on adjustable weighted parameters described below.

We therefore decided to create a new measure for progress in cancer research, called an E-Score (Figure 4), which is based on two primary components: the strength of the evidence and the ‘value’ of the treatment. E-Scores are cumulative scores that aggregate individual Pieces of Evidence over time. Each Piece of Evidence is weighted by the strength of the evidence (e.g. RCTs have greater weight than observational or retrospective studies) and by the potential stakeholder-specific value of the treatment, which stakeholders can designate using the value model described below. Other factors, such as effect size or drug class, can be used to adjust E-Scores based on stakeholder needs.

### Strength of the evidence

All evidence is not equal. Systematic reviews of primary data, which often include meta-analyses of RCTs and other trials, can often reach stronger conclusions than reports of individual clinical trials. This is because meta-analyses are often used for controversial topics where there are no definitive studies, and analysing pooled data from several smaller trials is necessary to confidently draw conclusions [11]. Some systematic reviews, such as Cochrane reviews, also specifically control for publication bias to ensure that no particular research is overrepresented [12]. Evidence from well-conducted RCTs is usually more robust than that from observational and retrospective studies. The first release of the Indicators will incorporate only publicly available data. Because most clinical trials with at least one site in the United States are now required by law to publicly report results to *ClinicalTrials.gov* within one year of completion, references to clinical trials in the Indicators’ database should be fairly complete. However, a recent study showed that only 22% of studies completed in 2009 met the requirement to report results [13].

E-Scores account for differences in evidence strength functionally and numerically by using an Evidence Hierarchy/Quality weight. Meta-analyses are the highest functional tier and can, as described below, override evidence produced by individual studies in certain circumstances. Nevertheless, meta-analyses do not receive the highest numerical weight when calculating E-Scores because doing so would artificially increase E-Scores for diseases with many controversies and small trials. Instead, in the current default settings, individual clinical trials receive the highest numerical weight, and both observational studies and meta-analyses receive lower weight. If the authors of the original trial report nominally significant p-values for the treatment effect but note that the side effects were so severe that the trial failed, we record the statistics from the trial in a Piece of Evidence but do not allow it to increment E-scores.

Some Pieces of Evidence are considered ‘group evidence’ when the treatment effect cannot be attributed to a single factor, for example when a study examines a drug class rather than individual drugs. We assign a fractional weight to results of these studies because it is unclear how much each individual drug contributes to the overall effect. If these individual effects are identified for pre-planned analyses, then we include the individual results. Some or all of the treatments tested as a drug class in one study might also be singly represented as Pieces of Evidence based on trials that tested the treatment individually.

With each data release, the analysts who curate the data revisit the existing evidence to determine how new Pieces of Evidence change their assessment of previous pieces. This procedure is described in detail in Appendix 2.

In brief, if a systematic review, for example a meta-analysis by the Cochrane Collaboration, concludes that the supporting evidence for a treatment in a certain context is not strong enough to support this treatment any longer, then the E-score algorithm disregards (though does not delete) all prior Pieces of Evidence for that particular treatment until further notice. If Pieces of Evidence prove to be invalid in this way, they become flagged and disqualified in the next data release, rather than decrease the E-Scores ‘in real time.’ This means that the entire cumulative E-Score curve for the corresponding cancer will be lower in the following data release, but the slope will never be negative. Additionally, the analysts can query the database and obtain statistics on how often treatments that showed significant effects in one or more trials were later re-assessed and found to be ineffective.

This procedure establishes an audit trail and ensures complete accountability regarding the underlying evidence, because no records are ever deleted from the database. It is, of course, fully possible that additional trials may reverse the assessment once again, or that improvements of a treatment lead to statistically significantly improved outcomes, in which case the analysts will re-assess the existing evidence. This procedure reflects the stepwise nature of progress in cancer science and provides a realistic picture of the many trials and studies that are often necessary to understand the benefit of a new treatment.

### Value Matrix

**Figure 5.**
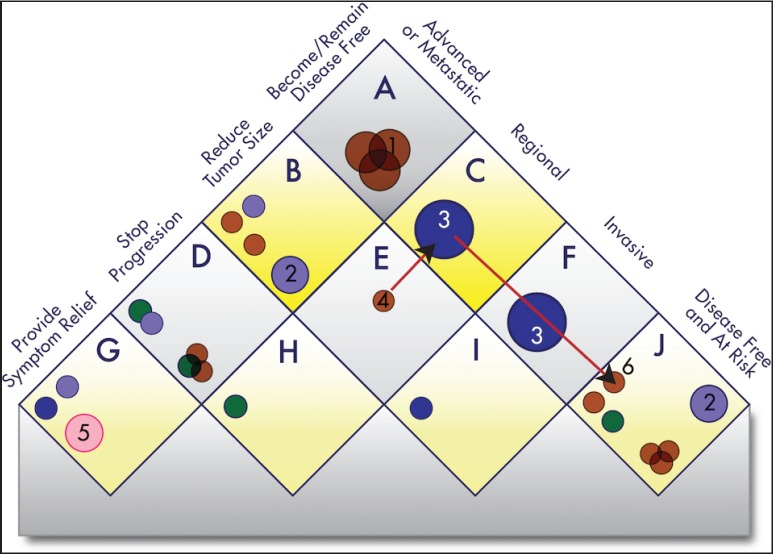
Value Matrix. The Value Matrix provides a framework for stakeholders to assign weight to different therapeutic goals. Each Piece of Evidence is classified in terms of disease state (right side of matrix) and one of four progressively more ambitious treatment goals (left side of matrix). Each circle represents a treatment. The colour of the circle denotes the treatment modality (surgery, blue; radiotherapy, green; chemotherapy, brown; immunotherapy, gray; group evidence (e.g. for a treatment class), purple; other, pink), and the colour intensity denotes the strength of the evidence (the darker the shade the stronger the evidence). Combination treatments and improved treatments have separate symbols. The size of the symbols denotes whether the treatment is currently offered to only a few patients (small), to most patients (medium), or to essentially all patients (large). For example, a chemotherapy treatment that strong evidence shows is highly effective for preventing a regional cancer from progressing to advanced disease and is the current standard of care would be represented as a large, dark brown circle in square ‘H’. See Appendix 3for a complete description of the categories and Appendix 4for the standard procedures to determine the circle size. The alphabetic square labels are for classification only and confer no value judgment. Stakeholders can assign each square differing weights to influence E-Scores according to their priorities. The numbered circles exemplify various scenarios: 1) curative combination chemotherapy in advanced cancer; 2) group evidence showing that, for example, ’radiotherapy’ works in this context, without further specification; 3) curative surgery in early-stage cancers; 4) neo-adjuvant chemotherapy; 5) palliative care; and 6) adjuvant chemotherapy.

The Continuous Innovation Indicators seek to combine scientific accuracy with ease of use. Figure 5 shows a graphical representation of the current value model, which stakeholders can use to assign weights to different treatment goals. The right side of the matrix represents the state of the disease, and the left side represents the goal of the treatment. For example, the top square of the resulting matrix indicates treatments that cure advanced disease.

Each new therapy (represented by a circle) can map to one or more squares on the matrix. If the goal of a treatment is, for example, to make the patient disease-free after a diagnosis of advanced or metastatic cancer, then it will be represented in the top square labeled ‘A’. If the goal is to stop progression of an advanced or metastatic cancer, then it will be represented in the square labeled ‘D’, whereas a treatment meant to keep patients with resected tumours disease-free will be represented in the square labeled ‘J’ (see Appendix 3for additional explanations).

By assigning a different value to each square of the value model, stakeholders can calculate E-Scores according to their priorities. For example, a stakeholder who is primarily interested in progress only of curative therapies may assign greater weight to squares ‘A’, ‘C’, ‘F’, and ‘J’ and relatively less weight to other squares. The resulting E-Scores would depict a different portrayal of progress than if all treatment goals were weighted equally (see Appendix 5 for further details regarding this methodology).

The shade of the circles correlates with the strength of the evidence, while the size indicates whether the treatment is offered to some patients (small symbol), most patients (medium), or essentially all patients (large). Targeted treatments that have strictly defined molecular eligibility criteria are represented by small circles if they constitute the standard of care for a small, defined group of patients. If additional evidence becomes available indicating that a targeted treatment benefits a larger share of the patient population, the corresponding circle ‘grows’ in the visual representations over time.

The first release of the Indicators will focus on the outcome of overall survival. Therefore, only treatments that have been shown to improve overall survival (fulfilling criteria for statistical significance after correcting for multiple testing) are plotted onto the matrix and included in the E-Score calculations. One advantage of our model compared to other efforts to track innovation is that it accounts for multiple treatment modalities (e.g. surgery, radiotherapy, and drug therapy). It further allows the analysts to create ‘treatment goal paths,’ which are depicted as red arrows. The value of neo-adjuvant treatment, for example, will often be producing a larger subpopulation of operable cancers and increasing survival, not by shrinking the tumour per se but by increasing surgical success rates. Thus, the treatments connected in a path work together to improve outcomes for patients, and including additional treatments in these paths can lead to overall outcome improvements that are larger than the sum of its parts. These synergies are rarely evident when a new treatment is introduced and often require a substantial number of additional studies. It is further possible that additional treatments interact with available treatments, for example when neo-adjuvant treatments might threaten to obscure nodal status and impair pathological staging [14]. The treatment goal path approach allows us to account for these complex interactions.

### Custom weights

Custom weights, the final component of E-Scores, include additional parameters to tailor E-Scores according to stakeholder priorities. Custom weights can factor in, for example, effect size (e.g. hazard ratio or difference in median survival between the treatment and the control group), drug class, and other variables of stakeholder interest.

### E-Score formula

**Figure 6.**
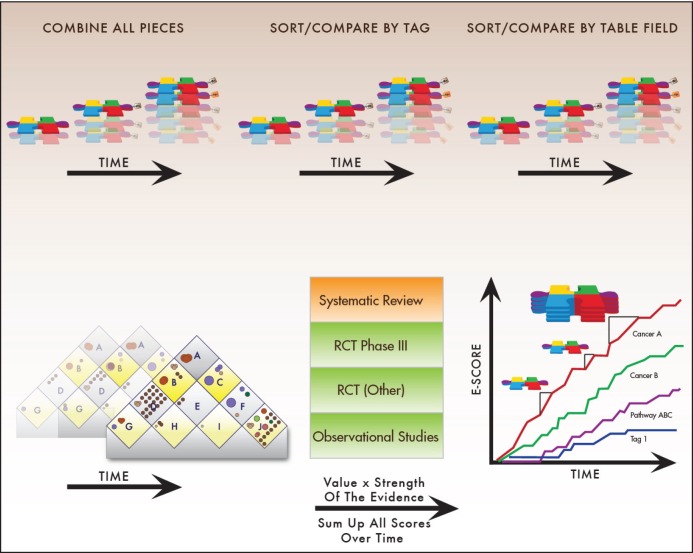
Evidence Scores explained. Tracking research progress requires a quantitative scoring mechanism. To accomplish this, the Continuous Innovation Indicators use E-Scores. Users can select which kinds of records they are interested in scoring (e.g. all lung cancer records, or only HER2-targeted metastatic breast cancer treatments, or only radiation therapies for early prostate cancer). They can also assign their own values to research progress toward specific therapeutic goals (e.g. some stakeholders may value progress toward curative therapies for advanced disease strongly compared to other goals, while others may assign greater relative value to treatments that provide symptom relief). E-Scores measure the number of Pieces of Evidence that support a treatment, adjusted by the strength of the evidence, value of the treatment goal to the user, and other user-assigned parameters, such as effect sizes. The results are displayed in a graphical format.

The mathematical formula for the E-Scores is: evidence x value weights x custom weights. Figure 6 summarises the algorithm that turns accumulating Pieces of Evidence into E-Scores. The process begins with a database query to select Pieces of Evidence for analysis based either on tags or on any other information that can be found through the relational database.

Once the relevant Pieces of Evidence have been identified, the algorithm calculates E-Scores using the Evidence Hierarchy, value weights from the Value Matrix, and any custom weights selected. The resulting curves go up when the evidence increases. Based on the value weights, some new treatments may lead to considerable increases in E-Scores within a short time period. More often, however, in accordance with the paradigm of continuous innovation in cancer research, gradual increases add up to larger increases over time.

Users may modify the weights of the Evidence Hierarchy, Value Matrix, and custom weights that underlie the algorithm to match their own values or priorities. The weights, in particular, are likely to differ among stakeholders. Because values can change over time, the scoring algorithm accepts several such matrices (for different points in time), using the scores from the closest preceding matrix.

## Results

We recognise that visualisation and quantification of progress in cancer research needs to be systematic and evidence-based to ensure its utility for stakeholders.

**Figure 7.**
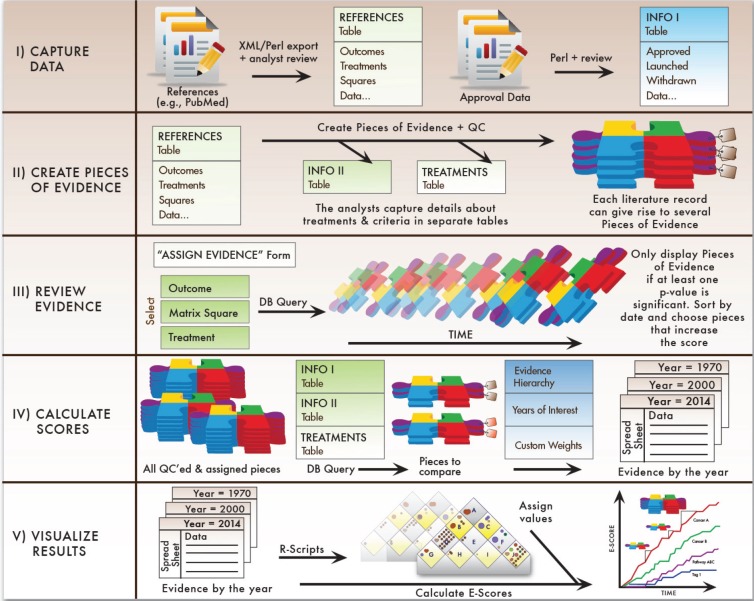
Summary of the Continuous Innovation Indicators approach. The Continuous Innovation Indicators comprise five stages. First, cancer treatment data are extracted from desired sources, reviewed by analysts, and stored in a table in a relational database. The relational design allows storage of multiple data types that are available for comparison based on end-user queries. Next, analysts systematically create distinct Pieces of Evidence as described above. Once all evidence has been curated, analysts query the database to review and assign evidence relevant for calculating E-Scores. The assigned Pieces of Evidence are the units of analysis for the algorithm, which calculates raw E-Scores for each year based on user-defined query parameters. Finally, the system adjusts scores based on the weights users assign to different therapeutic goals and creates a layered graphical output that allows users to view results at their desired level of detail.

The approach is detailed below and summarised in Figure 7.

- The first step is data capture. The Continuous Innovation Indicators allow users to import large sets of records from existing databases with ease. The first release will focus on results of clinical trials (e.g. all PubMed reports of Phase 2 and Phase 3 trials) and studies that have been identified by others as key findings, for example, all references cited in the National Cancer Institute (NCI) Physician Data Query (PDQ) guidelines. We also include references from the Cochrane Library. Because we envision this tool as a dynamic resource that improves over time, we will include additional references whenever necessary. To allow comparisons of drug approval dates between countries, we can also import data from Citeline Pharmaprojects® and other sources that contain information on the development, approval, and launch of new therapies. We aim to provide updated versions of the references about every three to four months.
- The data from the above references are, in their native format, not suitable for quantification, because each study may contain multiple outcome measures. Disaggregating them into distinct Pieces of Evidence facilitates compilation of a common body of knowledge that stakeholders can review, discuss, and revise.
- When new Pieces of Evidence have been created, they not only add to the existing evidence, but also may modify the net findings derived from the resulting body of evidence. Therefore, the system pulls up all Pieces of Evidence for the same treatment and square every time a new Piece of Evidence has been added so that the analysts maintaining the system can determine whether the E-Score should change and whether the validity and importance of older pieces need to be revised.
- Once the relevant data have been captured and checked for quality, the user can query the database to retrieve all the Pieces of Evidence that are relevant for a particular clinical context. Based on the available evidence, the Evidence Hierarchy, and optional custom weights, the system then generates raw scores for each year of interest.
- Finally, the evidence is weighted by the values from the Value Matrix. Experience with test audiences suggests that users may find it useful to plot the existing treatments onto the matrix before assigning the values to increase consistency between different raters. In addition, it may be useful to plot similar scenarios from different cancers so that users can learn from previous developments and set realistic priorities and expectations.

Appendix 5 contains several hypothetical scenarios to illustrate the effect of differing values on the E-Score results.

## Forward-facing flexibility

Flexibility is one of the greatest strengths of the Continuous Innovation Indicators. Indeed, the Indicators are designed to grow alongside of continuous progress in cancer research. The streamlined approach to integrating large amounts of new evidence and reassessing past evidence in the present context could, for example, be used to trigger alerts when new evidence emerges that substantially alters prior conclusions. Virtually all components of the Indicators can be adjusted and enhanced to serve evolving needs. Different outcome measures, data sources, and refined weighting systems can all be incorporated.

The first release of the Continuous Innovation Indicators will focus exclusively on studies that measure overall survival. Overall survival is a broadly accepted, well-defined, and commonly measured outcome and is therefore a logical starting point. The Indicators can easily incorporate additional outcome measures, such as progression-free survival, disease-free survival, or measures of mortality at the population level.

One outcome measure of great interest to many stakeholders of cancer treatment research is quality of life (QOL). Unfortunately, QOL is difficult to measure and interpret. A recent American Society of Clinical Oncology (ASCO) cancer research committee working group on pancreatic cancer tasked with defining clinically meaningful outcomes concluded that, while important, improvement in QOL should not be used as a primary endpoint for clinical trials because ‘current global quality of life questionnaires are not considered to be useful’ [15]. Instead, the working group recommended that future trials focus on specific disease-relevant symptoms. Indeed, none of the four disease-specific working groups included measures of QOL in their final recommendations for future trial designs. Until the research community reaches a clearer consensus on robust and meaningful measures of QOL, it is not feasible to incorporate QOL into the Continuous Innovation Indicators.

Others have noted a difference between efficacy and effectiveness of treatments: efficacy refers to the benefit of a treatment under ideal or controlled conditions, such as RCTs, and effectiveness refers to the benefit of a treatment under routine or ‘real-world’ conditions, such as treatment in community oncology settings [11, 16]. In the first release, the Indicators will be skewed—especially for drugs because only prospective studies are included—toward measuring progress in efficacy of cancer treatments. This is because most references currently in the Indicators database are RCTs or meta-analyses thereof. The Indicators are flexible enough, however, to include measures of effectiveness by adding new data sources. For example, references of comparative effectiveness studies and even real-world data from the ASCO’s Institute for Quality, Learning Intelligence Network for Quality (CancerLinQ) platform or comprehensive clinical cancer registries can be incorporated. The analysts can adjust the Evidence Quality/Hierarchy weighing scheme to include real-world data or observational studies as desired.

Incorporating new data sources can also mitigate the effects of publication bias. Publication bias refers to the greater likelihood of some studies (e.g. those with positive results) to be published over others (e.g. those with negative results). The Indicators may be somewhat influenced by publication bias because they incorporate broad selections of published literature. Others have identified strategies to control for such biases [17], some of which are used by systematic reviews that the Indicators rely on to resolve research controversies. Diversification of data sources to include ‘real-world’ data, such as from CancerLinQ or cancer registries, may be another effective strategy.

Many approaches have been developed to assess the quality of evidence and could be used to refine the Continuous Innovation Indicators Evidence Hierarchy. For example, the Grading of Recommendations Assessment, Development, and Evaluation (GRADE) working group developed a now widely used two-step approach to rate the quality of evidence on a four-tier confidence scale [18]. First, the initial level of confidence in study results is established: RCTs are ranked as ‘high confidence’ and observational studies as ‘low confidence’. Confidence ranks are then adjusted up or down based on the risk of bias and other factors. The result is that each study, regardless of its design, receives a rank (high, moderate, low, or very low) pertaining to the reviewer’s confidence in the study results. The Agency for Healthcare Quality and Research Evidence-based Practice Centers use a similar approach for evaluating evidence [19]. Such approaches can be adapted and incorporated into the Indicators scoring algorithm.

## Discussion

Evidence-based medicine is an important cornerstone of modern health care systems. Informed policymakers are more likely to invest in health than those without exposure to evidence regarding the benefits of existing and novel treatments. Under global fiscal constraints for health care systems, evidence about benefits and harms to individuals and populations become even more critical because it helps to inform investments about innovation and policies mediating access to novel treatments.

The Continuous Innovation Indicators can serve as an educational tool to support evidence-based investments by informing policymakers and the public about the stepwise nature and paths of cancer research progress. Publications about advances in research often focus on milestones and ignore the many iterative improvements of existing drugs, combination treatments, and multimodal treatment approaches. This leads to the false impression that cancer research progress occurs in great leaps. It is remarkable that the debate in public media is still dominated by black and white statements on whether or not we have made progress against cancer [20–22]. Focusing instead on objective reviews of the relevant bodies of evidence can lead to a more differentiated and useful review of progress in cancer treatments.

The Continuous Innovation Indicators do not offer clinical guidance, and they are not meant to replace existing treatment guideline publications. Further, the Indicators are not meant to replace current efforts by the Cancer Intervention and Surveillance Modeling Network (CISNET) [23] and other groups that conduct careful assessments of the impact of cancer treatments on overall survival at the population level. These careful and complex simulations are absolutely necessary to arrive at scientifically sound estimates of treatment effects in complex environments.

The E-Scores computed with our tool are not absolute values that quantify overall survival but instead measure the increasing evidence supporting new treatments against cancers of interest. Similar to other commonly used indices, their primary purpose is to track changes over time and between areas of interest, not to provide an absolute value that can be directly translated into survival estimates. At the core of the Indicators is a large database that allows analysts to carry out many other queries, for example to understand how many treatments have been deemed ineffective after their introduction, whether or not certain countries or regions approve new treatments faster than others, etc.

We hope that the disaggregated Pieces of Evidence approach will stimulate interactions among scientists to establish a body of knowledge of accepted evidence. While we do not expect the Continuous Innovation Indicators to ever substitute for carefully conducted systematic reviews, we hope that they will become useful to those in the field searching for a reference framework. By getting a faster overview of the state of the art, possible comparators, and unmet needs, we hope to be able to contribute to accelerating the important work of those who evaluate new treatments.

Continuous updates of the Indicators as a central resource hold the promise to create a resource for those in the field similar to the availability of a public genome-browser for those working in genomics [24]. We are currently assessing 12 solid tumours (breast, colon, endometrium, kidney, liver, lung, pancreas, prostate, skin [melanoma], stomach, rectum, and testes) for a first public data release in the first quarter of 2015. Full transparency and accountability are keys to success of this tool. Access to all underlying data is critically important for qualified users to be able to customise the tool according to their own needs. We encourage organisations who want to partner with PACE to contact the corresponding author.

We hope to contribute to ongoing discussions about value in cancer care by highlighting the critical importance of stepwise innovation. Furthermore, identifying unmet needs will stimulate discussions of greater societal benefits of new treatments. The Indicators are thus meant to complement, not replace, ongoing efforts to discuss cost-effectiveness of new treatments. Value in cancer care is a very complex concept that will likely require multiple efforts and approaches to define and translate into concrete action by policymakers and others who set priorities for cancer treatment developments. The Continuous Innovation Indicators are meant to become one piece in this complex puzzle.

Finally, we hope that components of our approach, such as the Value Matrix, will be useful in supporting policy discussions as outlined in Appendix 6.

## Conclusions

The PACE Continuous Innovation Indicators provide a novel tool to:

1. Establish a relational database that allows execution of queries such as:
  - Number of times we learned more about treatments after they were introduced
  - Number of times treatments did not work out as planned
2. Keep track of progress in a flexible framework that allows the analysts to incorporate relevant new evidence in real time and to quickly determine the impact of this new evidence on the assessment of the available body of relevant evidence
3. Gain a better understanding of the complex evolution of value in cancer treatment
  - Visualise how stepwise progress contributes to significant progress against cancer over time, including the synergies of combination and multimodality therapies
  - Illuminate the extent of progress and allow for comparisons across anatomical sites, treatment approaches, molecular subtypes, and other stratifying variables
4. Establish a map of remaining unmet needs in the treatment of cancer
5. Illustrate the potential impact of cancer-policy reforms

Differentiated analyses based on values provided by various stakeholders will help the cancer policy field to obtain accurate representations of the complex, stepwise progress against different cancers over time. We encourage organisations who want to partner with PACE to contact the corresponding author. We envision partnerships and collaborations to support educational efforts, identification, and illustration of policy goals, and work in the field of health technology assessments. We will not make this tool available to individuals or organisations for the purpose of deriving treatment recommendations.

**Table:** List of acronyms

Acronym	Definition
ASCO	American Society of Clinical Oncology
CancerLinQ	Learning Intelligence Network for Quality
CISNET	Cancer Intervention and Surveillance Modeling Network
GRADE	Grading of Recommendations Assessment, Development, and Evaluation
HER2	Human epidermal growth factor receptor 2
NCBI	National Center for Biotechnology Information
NCI	National Cancer Institute
PACE	Patient Access to Cancer care Excellence
PDQ	Physician Data Query
QOL	Quality of life
RCT	Randomised controlled trial

## Figures and Tables

**Figure 1.**
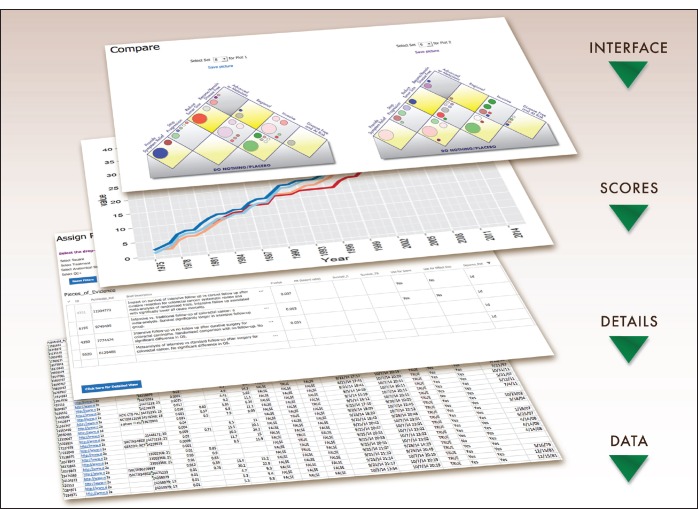
Overview of the Indicator layers. The PACE Continuous Innovation Indicators provide a layered, user-friendly interface capable of displaying a range of information from high-level summary statistics to details at the individual study level. The primary interface allows the user to select results plotted onto the Value Matrix, an innovative platform described herein for weighting the value of treatments based on their therapeutic goals. The next layer provides comparison graphs of cancer research progress as Evidence Scores (E-Scores), a novel quantitative measure of cumulative progress. Advanced users can customise the value weights of therapeutic goals, and indeed of all input parameters, to generate results aligned with their own priorities. Finally, for complete transparency, analysts can access the underlying data tables to obtain results of individual clinical trials and links to the original references that are used to compute summary scores.

**Figure 2.**
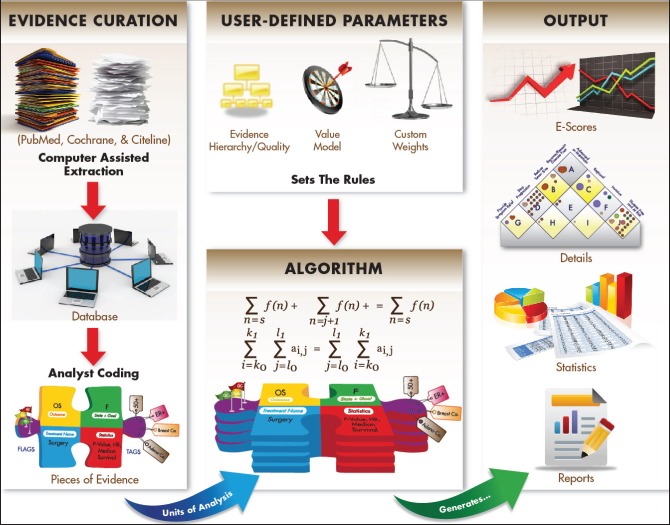
Schematic overview of the Continuous Innovation Indicators method. At the heart of the Indicators is an algorithm that operates under a set of user-defined parameters to generate output measures from Pieces of Evidence, the units of analysis. Pieces of Evidence are derived from a variety of sources, including clinical trial records, meta-analyses, and historical references, which are first extracted into a relational database and then coded by trained analysts according to a standard protocol. Three user-defined parameters set weights for the analysis: The Evidence Hierarchy/Quality rating determines the weight assigned to different types of references (e.g. randomised controlled trials, meta-analyses, systematic reviews), the Value Matrix determines the weight assigned to achieving various therapeutic goals (e.g. halting progression of advanced disease, shrinking a localised tumour), and optional custom weights account for other factors of interest (e.g. effect size, therapeutic modality). Based on the selected parameters and available evidence, the algorithm generates E-Scores, a novel measure of cumulative progress over time. Other outputs include a graphical representation of evidence on the Value Matrix, summary statistics, and detailed source information.

**Figure 1.**
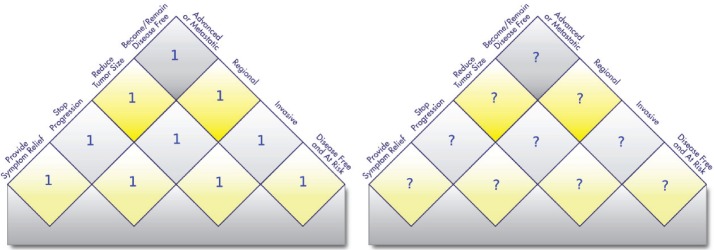
Do equal value weightings (left side) make sense? Should value weightings differ by cancer and/or stakeholder? Currently, it appears that new treatments are valued equally regardless of where they map onto the Value Matrix (each square valued at ‘1’, left matrix). The Value Matrix allows users to assign their own weights to potential treatment advances at any position in the matrix (right side). This allows evaluation of progress in cancer research in any value-system framework and could potentially support policy interventions to direct more research to current unmet needs.

**Figure 2.**
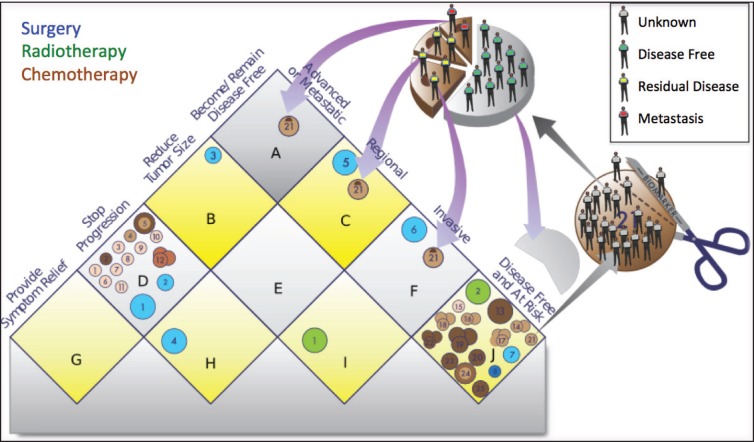
Disease-tracking biomarkers could have a substantial impact by identifying at-risk patient subpopulations. If biomarkers could be used to identify individuals with circulating tumour cells or residual disease, then at-risk individuals within the apparently disease-free population might be identified and treated with an appropriately targeted drug. This would push existing treatments into higher levels of the matrix and thus constitute very substantial progress in cancer research.

## Technical appendices

1. Sources of variability, strategies to maximise consistency, and estimates of remediation needs
2. Procedure for assigning Pieces of Evidence to calculate E-Scores
3. Detailed explanation of Value Matrix square assignments
4. Procedure for determining circle size of treatments on the Value Matrix
5. Values matter: the effect of value weights on E-Scores
6. Two examples of ways in which the Indicators can support policy discussions

## Appendix 1. Sources of variability, strategies to maximise consistency, and estimates of remediation needs

**Table:**

Step	Specific task	Source of possible variability	Strategies to maximise consistency	Records requiring remediation^1^
I) Data capture	Select literature references for data abstraction.	Analysts may select different references.	Analysts review common sets of references from standard sources. Computer-assisted extraction with Perl minimises data entry errors.	<1%
I) Data capture	For each reference, determine whether the outcome of interest was measured, which treatments were tested, and which squares apply for the goals of tested treatments.	Analyst interpretations of treatment names, goals, and outcome measures may differ. For example, many treatments and treatment combinations have synonyms. There are multiple possible names for tests of new dosages or sequences of combination therapy.	Blind duplicate reviews by multiple analysts increase consistency. An alias table for treatment names matches synonyms and links combinations and treatment improvements or modifications, such as dosage or duration.	<5%
II) Create Pieces of Evidence	Create separate Pieces of Evidence for each applicable treatment and square for a given reference.	Analysts may interpret references differently or overlook multiple treatments or squares that apply for a single reference.	Blind duplicate reviews by multiple analysts ensure that at least one Piece of Evidence is created for each treatment tested in each square in all references.	<1%
II) Create Pieces of Evidence	Accurately capture relevant statistics and apply data tags and flags for each Piece of Evidence.	Analysts may overlook relevant information or make typographical errors.	Blind duplicate reviews by multiple analysts increase consistency. Only fully complete and accurate Pieces of Evidence pass quality control by a third analyst.	<5%
II) Create Pieces of Evidence	Quality control review to ensure accuracy and completeness of Pieces of Evidence.	Quality control reviewer may overlook important information.	Quality control reviewers document the reasoning for their decisions, and a tertiary reviewer verifies the accuracy of quality control.	<1%
III) Review evidence	Determine which Pieces of Evidence increment E-Scores.	Analysts may overlook meta-analyses or definitive RCTs that refute prior significant findings.	Analysts document the reasoning for their decisions, and a secondary analyst reviews selections for concurrence.	<5%
III) Review evidence	Determine which Pieces of Evidence are used to calculate effect sizes.	Analysts may overlook caveats such as extensive crossover between treatment arms and anomalous or inverted statistics.	Analysts document the reasoning for their decisions, and a secondary analyst reviews selections for concurrence.	<5%
IV) Calculate scores	Select parameters for database query.	Analysts may select different parameters.	Parameters are deliberately adjustable to calibrate results to differing values and priorities.	0%
V) Visualise results	Determine the size of each circle that represents how widely a treatment is used in a particular context (disease and square).	Analysts may use different sources of information about treatment usage and may interpret the information differently.	Analysts document the data sources used and the reasoning for their decisions. A secondary analyst reviews each circle size assignment for concurrence.	<5%

1 These values reflect the proportion of records requiring remediation after implementation of the strategies to maximise consistency. Overall, about 15–20% of records require some type of remediation over the course of the entire process, including during the quality control processes described in this table.

## Appendix 2. Procedure for assigning pieces of evidence to calculate E-Scores

**Goal:** To determine which Pieces of Evidence should increment the E-Score.

**Introduction:** The evidence that a particular treatment improves overall survival is constantly changing. It is critical to re-assess the effectiveness of a treatment in light of additional information. The Continuous Innovation Indicators were designed to account for the fact that science is always evolving. The Innovation Indicators platform allows analysts to determine which Pieces of Evidence are used to calculate E-Scores by setting the score flag to either YES or NO. Pieces of Evidence with the score flag set to YES are used to increment the E-Score, and Pieces of Evidence with the score flag set to NO remain in the database but do not affect the E-Score. The platform is flexible in that the original value of YES or NO for a Piece of Evidence score flag can later be changed in light of superior evidence to the contrary. Evidence that is trumped by superior studies is never removed from the database, but is disallowed for the E-Score calculation, with the potential to reappear if sufficient data is present. Future research may prompt re-evaluations of evidence that would utilise the entire history of research on the particular treatment.

When setting score flags, analysts are able to view all Pieces of Evidence over time for a treatment in a given square in either one disease or across multiple diseases. Such a broad, chronological perspective allows analysts to visualise stepwise progress and facilitates the application of hindsight to refute past positive results based on results of more recent, superior studies. It also allows analysts to better understand how treatments are used across diseases and how advances in one disease are sometimes learned from experiences in another. The procedures described below assume the analyst considers Pieces of Evidence from a given square, treatment, and anatomical site.

**Procedure for setting the score flag for treatments with a single Piece of Evidence:** Many of the treatments in the database have only one associated Piece of Evidence. In general, the score flag is set to YES for a study demonstrating that a treatment significantly improves overall survival (p-value is <0.05). There are a few caveats for when the p-value is < 0.05, but the score flag should be set to NO. Setting the score flag to NO means the Piece of Evidence is disallowed for a reason specified by the analyst, including:

- If the authors of the study or meta-analysis state that their nominally significant p-value (<0.05) is statistically non-significant after correction for multiple testing, then set the score flag to NO.
- If the p-value is < 0.05, but the treatment toxicity is so severe that the authors state that the treatment should not be used, then set the score flag to NO.
- If the treatment is significantly (p < 0.05) worse than the control treatment, then set the score flag to NO.

Three other possibilities may also arise when setting the score flag for treatments with a single Piece of Evidence:

- If the p-value is >0.05, then the score flag is left BLANK and does not contribute to the E-Score.
- If the p-value = 0.05 and the authors do not clearly state that the results are significant, then set the score flag to NO.
- If the p-value is not provided, but the authors state that the statistics are significant or the confidence interval for the hazard ratio does not cross 1, then set the score flag to YES.

**Procedure for setting the score flag for treatments with multiple Pieces of Evidence:** There is an extra consideration for treatments with multiple Pieces of Evidence when deciding whether the score flag for an individual Piece of Evidence should be set to YES. It is possible that the results from one Piece of Evidence are so superior that they override or cancel out conflicting results from earlier Pieces of Evidence. Evidence accumulates over time, and results must be re-evaluated within the context of more recent or more rigorous studies. For example, a meta-analysis demonstrating a significant negative treatment effect of a drug would ‘trump’ smaller individual studies that showed a positive treatment effect. There are two considerations when deciding if a Piece of Evidence is sufficient to overrule the findings of another Piece of Evidence:

1. The Pieces of Evidence must be similar in their treatment protocols and patient populations. Scientists frequently study drugs in different strengths, combinations, delivery routes, and patient populations; the studies must be similar enough to compare the treatment effects.
2. To overrule other study results, the Piece of Evidence must also have a superior study design. Drug trials differ in their strengths and limitations, with more reliable trials having large sample sizes, robust statistical analyses, and well-designed study protocols. To override a prior finding, a clinical trial must be a large, well-designed RCT specifically designed to settle a treatment controversy. Systematic reviews such as those provided by the Cochrane collaboration are also specifically designed to address controversies regarding the effectiveness of a treatment in a particular clinical setting.

To set the score flag for treatments with multiple Pieces of Evidence, follow the procedure above for treatments with a single Piece of Evidence, with the following additions:

- Treatments used in different drug combinations and/or patient populations all individually increase the evidence that a given treatment works and we set the score flag to YES if the p-value is <0.05.
- Individual positive trials leading to a positive systematic review are all included in the Evidence Score, as this is a measure of accumulated evidence over time. Future functionality of the Indicators will allow the user to disallow meta-analyses if all contributing trials have already been counted individually.
- The data management approach of the Indicators can readily incorporate new evidence that a treatment significantly improves overall survival; every time new study results are available, the database pulls up all relevant previous records to allow the analyst to review the impact of the new evidence.

**Challenges and solutions in setting the score flag for treatments with multiple Pieces of Evidence:** Some potential challenges when setting score flags for treatments with multiple Pieces of Evidence are described below.

**Table:**

Challenge	Solution
There are multiple Pieces of Evidence for a treatment that all demonstrate a significant increase in overall survival; each Piece of Evidence is unique in that the treatment is used with a different drug combination or a different patient population.	Because the Pieces of Evidence are unique, each Piece of Evidence increases the E-Score on its own and each score flag should be set to YES.
There are multiple Pieces of Evidence for the same treatment under the same conditions (i.e., identical treatment and control arms and patient populations); however, some Pieces of Evidence demonstrate a significant increase in overall survival and others demonstrate a non-significant effect. None of the Pieces of Evidence represents a meta-analysis or large RCT designed to settle a controversy.	Set score flags for all Pieces of Evidence with p < 0.05 to YES.
There are multiple updated versions of the same systematic reviews examining a treatment in the same clinical setting, and all reviews show a significant effect for overall survival.	Only the score flag for the most recent review is set to YES; the score flags for the previous reviews are set to NO.
A Piece of Evidence concludes that a treatment significantly decreases overall survival.	The score flag of this record is set to NO. The analyst must determine if this Piece of Evidence negates others that demonstrate a significant increase in overall survival. If there are other Pieces of Evidence that indicate significant treatment benefits AND if the negative study was a meta-analysis or a large RCT specifically designed to settle a controversy, then this trumps any positive study results. In this case, all Pieces of Evidence with significant p-values are disallowed and we set their score flags to NO.
One Piece of Evidence is a systematic review or large meta-analysis or an RCT specifically designed to settle a controversy. This Piece of Evidence demonstrates a non-significant treatment effect on overall survival.	Because the meta-analysis or large RCT is non-significant, the score flag is left BLANK. If there are other Pieces of Evidence that indicate significant treatment benefits, then their score flags should be set to NO, provided study characteristics are comparable to those examined by the meta-analysis.
One Piece of Evidence is a systematic review or large meta-analysis or an RCT specifically designed to settle a controversy. This Piece of Evidence demonstrates a significant improvement in overall survival. Other Pieces of Evidence have reported a non-significant effect on overall survival.	A meta-analysis or large RCT demonstrating that a treatment significantly increases overall survival would negate an individual study with a non-significant p-value for overall survival. In the database, the score flags for the systematic review and any positive trials are set to YES and the score flags for any non-significant trials are left BLANK.

## Appendix 3. Detailed explanation of Value Matrix square assignments

**Table:**

Square	Type of study/treatment	Comment (based on pilot studies measuring overall survival)
A	Treatments that cure advanced and metastatic cancers.	Combination chemotherapy for metastatic cancer, which can be discontinued after treatment, places in this square. Treatments that turn cancers into chronic diseases that require further treatment with acceptable side effects may be placed here too.
B	Pharmacological or radiological treatments in advanced cancers that can increase the number of operable cancers or that reduce the tumour with a measurable effect on the quality of life.	Most treatments reduce the size of the tumour, as measured in the response rate, but it is not always clear whether that reduction persists or makes any difference for the patients. Included here are only trials with a clear downstaging goal, multimodal treatments, or trials in which the reduction of the tumour size had other measurable benefits for the patient. Treatments that can reduce the tumour size until they are only detectable by molecular methods may be placed here or in the ‘4’ square depending on whether life-long treatment to keep the tumour from growing back is an acceptable option.
C	Surgical removal of the tumour and lymph nodes and other regional affected structures.	Treatments are included here if the evidence is based on regional disease only, not a mix of invasive and regional cancers.
D	Treatments that can halt the progression of advanced/ metastatic cancers.	Most new pharmacological treatments ’enter’ the matrix in this square, because they are first tested in second- or third-line settings against advanced cancers.
E	Neo-adjuvant treatments that increase the surgical cure rate for regional cancers.	Not all trials are designed to isolate the effects of neo-adjuvant versus adjuvant treatment. Downstaging goals are not always clearly stated and measured.
F	For most cancers, surgical resection with curative intent will be used to treat most of these patients.	If trials contain a mix of node-positive and node-negative patients, then the model assigns this square unless the groups were tested separately in pre-planned tests. Therefore, this square contains some studies that contain node-positive and node-negative early-stage and regional cancers.
G	Treatments (pharmacological, surgical, other non-pharmacological) that aim to relieve symptoms; earlier initiation of palliative care.	Extending survival by focusing on symptom relief is rare, because most trials focus on extending overall survival with acceptable side effects. Relatively few trials address these interventions.
H	Treatments that can halt the progression of mostly inoperable regional cancers.	The use of the terms ‘regionally advanced’ and ‘advanced’ is not clear-cut in the trial literature. Our model applies a two-year survival threshold in the control group as a limit to distinguish between aggressive ‘regional’ and ‘advanced or metastatic’ cancers.
I	Neo-adjuvant treatments that increase the surgical cure rate for localised cancers.	Not all trials are designed to isolate the effects of neo-adjuvant versus adjuvant treatment. Downstaging goals are not always clearly stated and measured.
J	Adjuvant treatments. Continued treatment for tumours that can be shrunk to levels that can only be detected by molecular methods. Also includes screening of high-risk populations.	Many trials of new treatments for operable cancers have been conducted in recent decades.

## Appendix 4. Procedure for determining circle size of treatments on the Value Matrix

**Goal:** To graphically depict the prevalence of the use of various cancer treatments within a given square of the Value Matrices.

**Theory:** Each treatment in the Innovation Indicators database is represented as a circle on the Value Matrix. The size of the circle depicts the relative prevalence of the use of the treatment for that matrix square for that Value Matrix. Value Matrices may represent a cancer anatomical site, a treatment strategy (e.g. a targeted therapy or immunotherapy Value Matrix), or another treatment context defined by the user, as well as the same parameters at different points in time.

There are three circle sizes. The largest circle size is assigned to treatments that are used in almost all patients within a particular matrix square for a given Value Matrix. If a treatment is used for the majority of patients for a particular matrix square, then it would be assigned a medium circle size. The smallest circle size indicates that the treatment is one of a number of options for a particular treatment context for a given matrix square. Most treatments in our current data set have been assigned a small circle size.

### General Considerations

- Circle sizes are assigned to every treatment in the database, for every matrix square the treatment appears in, and for each relevant treatment context. For example, the same treatment may be assigned a large circle size for a given square in the Value Matrix for one anatomical site and a small circle size in a similar matrix square for a different anatomical site that sees less use of the treatment. The same treatment may also appear within another Value Matrix for a treatment strategy. In all cases, we have to assess the proportion of patients who receive a certain treatment in the context of a given matrix square for the relevant Value Matrix.
- Possible public sources we can use to determine the prevalence of the use of a treatment within a matrix square include 1) the National Cancer Institute (NCI) Physician Data Query (PDQ) (Health Professional Version), and 2) the American Cancer Society *cancer.org* web pages on cancer treatment. Partnering with other organisations that release treatment guidelines would make additional resources available for this purpose. Real-world data such as the NCI Surveillance, Epidemiology, and End Results-Medicare data linkage, or the American Society of Clinical Oncology CancerLinQ data are also possible sources that would provide further information about actual treatment usage.

### Circle Assignment Criteria

- A treatment is assigned a large circle size if it is the treatment of choice for essentially all patients with the treatment context under consideration for the relevant matrix square, with few exceptions. Generally, the only patients who are not recommended to receive the treatment are those who cannot tolerate the treatment. This exclusion criterion should be rare for the largest circles.
- A treatment is assigned a medium circle size if the treatment is likely to be recommended for the majority of patients for the treatment context under consideration within the relevant matrix square. Examples of situations in which a treatment may be designated as medium circle size include:
  - Treatments that are used to treat most patients, but other treatment options are available and are sometimes used in preference to it.
  - Treatments usually recommended for patients who meet certain selection criteria, such as histological features, anatomical features, or the presence of a particular genetic marker, but only if the majority of patients for the relevant matrix square meet the selection criteria.
- All treatments that are not used in the majority of patients for a given matrix square for the treatment context under consideration are designated as a small circle size.
- For quality control, a second analyst should examine the assignments and note whether he or she agrees or disagrees. Any disagreements about the assignments should be resolved between the analysts or by a third arbiter.

### Procedure

1. Search for relevant information on treatment usage. Look for information in the context of the relevant matrix square. For example, if the treatment is used for adjuvant therapy and also to treat advanced disease, but the context of the matrix square is to remain disease-free, then look for relevant information on adjuvant therapy only.
2. Using the general considerations from the section above, assess whether the treatment is used in essentially all patients in the context of the appropriate matrix square (large circle), in the majority (medium circle), or in fewer than the majority of patients (small circle).
3. Document your reasons for the circle size assignment you make, based on the information you find.

## Appendix 5. Values matter: the effect of value weights on E-Scores

This chart illustrates the influence of the matrix values on the E-Scores. We compare four different scenarios: Panel I shows a model in which the user does not value any treatment goal higher than any other. In II, a simple model has been applied in which treatments further ‘up’ in the matrix get a higher score. Panel III shows the effect of assigning high values to treatments for advanced and metastatic cancer, while panel IV shows the results for a model that puts a high value on treatments that keep the patients disease-free (e.g. adjuvant treatments and treatments targeted toward high-risk populations).

The four lines indicate E-Scores for four anonymised cancer sites from our current data set of common cancers for the period between 1985 and 2014.

The purple line represents a hard-to-treat cancer for which few effective treatments have been identified so far. It does poorly in each scenario. The red line represents a cancer with availability of both early- and late-stage treatments. The blue line represents a cancer with considerable successes in early-stage and adjuvant treatment, while the green line represents a cancer with comparatively more progress at advanced stages.

Below is an example of commented Perl code that we use to plot these charts from database exports (file paths, cancer names, and some internal variables deliberately obscured):

#!/usr/bin/perl -w

use strict;
use Text::CSV; #on CPAN
use Excel::Writer::XLSX; #on CPAN

#hashes to store data

my %EqualSquareWeight; # key: Square; value: Score for this Square 
my %DefaultSquareWeight; #key: Square; value: default score for this Square

my %EqualEvidenceWeight; #key: PubType; value: Score for this type of Evidence
my %DefaultEvidenceWeight; #key: PubType; value: Score for this type of Evidence

my %TotalEvidenceScore_I; #key: anatomical site; value: total score so far
my %TotalEvidenceScore_II; # second hash for comparison scenario

my %PublicationDate; #key: PMID; value: Date of Publication mm/dd/yyyy
my %EvidenceType; #key: PMID; value: type of evidence

my @CancerTypes = (‘Cancer1’, ‘Cancer2’, ‘Cancer3’, ‘Cancer4’); #anonymized cancers for publication

#years of interest for the charts
my @YearsOfInterest = (1985 .. 2014);

#read matrix value files
open (EQUALMATRIXWEIGHTS, ‘Equal_Matrix.txt’) or die “\nCould not find the equal weights file!\n”;

while (<EQUALMATRIXWEIGHTS>){
   chomp ($_);
   my @equalweights = split (/\t/, $_);
   $EqualSquareWeight{$equalweights[0]}=$equalweights[1]; 
}
close EQUALMATRIXWEIGHTS;

open (DEFAULTMATRIXWEIGHTS, ‘Default_Matrix.txt’) or die “\nCould not find the default weights file\n”;
while (<DEFAULTMATRIXWEIGHTS>){
   chomp ($_); 
   my @defaultweights = split (/\t/, $_);
   $DefaultSquareWeight{$defaultweights[0]}=$defaultweights[1]; 
}
close DEFAULTMATRIXWEIGHTS;

#read in evidence hierarchy file (this version has a score for each evidence type)

open (DEFAULTEVIDENCEWEIGHTS, ‘Default_Evidence.txt’) or die “\nCould not find the default evidence File!\n”;
while(<DEFAULTEVIDENCEWEIGHTS>){ 
   chomp($_); 
   my @defaultEviWeights = split (/\t/, $_);
   $DefaultEvidenceWeight{$defaultEviWeights[0]}=$defaultEviWeights[1];
}

#go through the list of publication dates and create hash of publication dates per PMID and a hash of Evidence types per PMID

my $csvAllRefs = Text::CSV->new ({ binary => 1}) # should set binary attribute.
or die “Cannot use CSV: “.Text::CSV->error_diag ();

open my $fh3, “<:encoding(utf8)”, “Publication_dates_master_list.csv” or die “Publication_dates_master_list.csv: $!”;

while (my $RefRow = $csvAllRefs->getline ($fh3)) { 
   $PublicationDate{$RefRow->[0]}=$RefRow->[1]; $EvidenceType{$RefRow->[0]}=$RefRow->[2];
}

# --- Go through the PoE export file and extract relevant fields ---

my $csvAllPoEs = Text::CSV->new ({ binary => 1 }) # should set binary attribute. 
or die “Cannot use CSV: “.Text::CSV->error_diag ();

open my $fh4, “<:encoding(utf8)”, “PoEs.csv” or die “PoEs.csv: $!”;

while (my $PoERow = $csvAllPoEs->getline ($fh4)) {

   my $GroupEvidenceWeight = 1; #all scores get a group weight of one unless the flag is “on”

   unless ($PoERow->[28] eq ‘Yes’) {next;} #continue only with those PoEs that are set to “Yes” in the “use for score” flag column

   my $recordYear = $PublicationDate{$PoERow->[1]}; if ($recordYear){$recordYear =~ s/\d+\/\d+\///g;} # convert pubdate to year only

  if ($PoERow->[17] eq ‘TRUE’){$GroupEvidenceWeight = 0.2;} # group evidence receives a lower weight because the individual treatment
contribution is less clear

  $TotalEvidenceScore_I{$PoERow->[4]}{$recordYear}=$TotalEvidenceScore_I{$PoERow->[4]}{$recordYear} +
  ($DefaultSquareWeight{$PoERow->[7]} * $GroupEvidenceWeight * $DefaultEvidenceWeight{$EvidenceType{$PoERow->[1]}}); #sum 
up E-scores for each year first scenario
  $TotalEvidenceScore_II{$PoERow->[4]}{$recordYear}=$TotalEvidenceScore_II{$PoERow->[4]}{$recordYear} +    
  ($EqualSquareWeight{$PoERow->[7]} * $GroupEvidenceWeight * $DefaultEvidenceWeight{$EvidenceType{$PoERow->[1]}}); #sum up 
E-scores for each year second scenario

}

# ------- End of Evidence Gathering Section --------
# Create Excel output file

my $workbook = Excel::Writer::XLSX->new (‘Results_date.xlsx’);
die “Problems creating new Excel file: $!” unless defined $workbook;

# -------------- I --------------
#create a worksheet for the first scenario
my $worksheet_I = $workbook->add_worksheet(‘I’); 
my $rowcounter_I = 0;

$worksheet_I->write($rowcounter_I, 0, “Cancer type”); #write header
my $headlinecounter_I = 1; 
foreach(@YearsOfInterest){
  $worksheet_I->write($rowcounter_I, $headlinecounter_I++, $_);}

#sum up data for each year and write data
$rowcounter_I++;
my $columntracker_I;
foreach my $outputCancer_I (@CancerTypes){ 
   my $columncounter_I = 1; 
   my $outputScore_I = 0;
   $worksheet_I->write($rowcounter_I, 0, $outputCancer_I);

   foreach my $resultYear_I (@YearsOfInterest){ 
      $outputScore_I = $outputScore_I + $TotalEvidenceScore_I{$outputCancer_I}{$resultYear_I}; 
      $worksheet_I->write($rowcounter_I, $columncounter_I++, $outputScore_I);
   } 
   $rowcounter_I++;
   $columntracker_I = $columncounter_I;
}

#create chart
my $chart_I = $workbook->add_chart (type => ‘line’, embedded => 1);

## configure chart
my $maxcolumnnumber_I = scalar(@YearsOfInterest);

for(my $sitecounter=0; $sitecounter<@CancerTypes; $sitecounter++){ 
   my $tempcounter = $sitecounter+1; 
my $CancerDisplayName_I = “Cancer_” . “$tempcounter”;
  $chart_I->add_series(
  name => $CancerDisplayName_I, 
categories => [ ‘I’, 0, 0, 1, $maxcolumnnumber_I ], 
values => [ ‘I’, $sitecounter+1, $sitecounter+1, 1, $maxcolumnnumber_I ],);}

$worksheet_I->insert_chart (‘B15’, $chart_I);

# -------------- II ----------------
#create a worksheet for the second scenario, then same as above
my $worksheet_II = $workbook->add_worksheet(‘II’);
my $rowcounter_II = 0;

$worksheet_II->write($rowcounter_II, 0, “Cancer type”);

my $headlinecounter_II = 1;
foreach(@YearsOfInterest){ 
   $worksheet_II->write($rowcounter_II, $headlinecounter_II++, $_);}

$rowcounter_II++;
foreach my $outputCancer_II (@CancerTypes){ 
   my $columncounter_II = 1;
   my $outputScore_II = 0; 
   $worksheet_II->write($rowcounter_II, 0, $outputCancer_II);

   foreach my $resultYear_II (@YearsOfInterest){ 
      $outputScore_II = $outputScore_II + $TotalEvidenceScore_II{$outputCancer_II}{$resultYear_II};
      $worksheet_II->write($rowcounter_II, $columncounter_II++, $outputScore_II);
   }
   $rowcounter_II++;

}
my $chart_II = $workbook->add_chart (type => ‘line’, embedded => 1);

## configure chart
my $maxcolumnnumber_II = scalar(@YearsOfInterest);
for(my $sitecounter=0; $sitecounter<@CancerTypes; $sitecounter++){ 
   my $tempcounter = $sitecounter+1;
   my $CancerDisplayName_II = “Cancer_” . “$tempcounter”;

   $chart_II->add_series( 
   name => $CancerDisplayName_II, 
   categories => [ ‘II’, 0, 0, 1, $maxcolumnnumber_II ],
   values => [ ‘II’, $sitecounter+1, $sitecounter+1, 1, $maxcolumnnumber_II ],);}

$worksheet_II->insert_chart (‘B15’, $chart_II);

## Appendix 6. Two examples of ways in which the Indicators can support policy discussions

### Policy discussion I—incentives

Many individuals and organisations have expressed frustration with the ‘incremental’ advances in cancer research of the past [1–3], but few efforts have been undertaken to understand the origin of these ‘incremental’ steps and to identify incentives that may sustain them.

The PACE Continuous Innovation Indicators Value Matrix can help users to visualise what incentives may be necessary to drive innovation, optimise treatments, and to systematically combine treatments to achieve higher treatment goals. In the current environment (left side of Figure 1), new treatments can ‘enter’ the Value Matrix in any square. Although most people would likely agree that we want to encourage aiming ‘as high as possible’ in the matrix, there is no roadmap outlining concrete incentives to actually do so. This is indicated in Figure 1 by assigning an equal weight of ‘1’ to each square. Similarly, after a drug has been approved, there is a public interest to obtain as much information as possible about additional uses of the drug in other squares.

The current incentive model of cancer research and development (R&D) may contribute to a lopsided clinical trial landscape (as pointed out by Budish *et al* [4]): most drugs ‘enter’ the value model in the ‘D’ square of our matrix. Most of these trials are sponsored by industry. Conversely, trials of additional uses, which usually take longer to conduct (especially trials on the right side of the matrix), are mainly sponsored by academic institutions.

There are few incentives for systematic analyses of, for example, neo-adjuvant versus adjuvant effects of a drug. Because there seem to be no clearly defined incentives to drive this better understanding, the effect of drugs in additional squares (in addition to the square where they first entered) are often very loosely defined when measured in clinical trials (e.g. statements such as this, ‘The lower rate of pneumonectomy (15% versus 25%) and of resections greater than standard lobectomy (28% versus 52%) performed in the chemotherapy plus surgery arm compared with the surgery alone arm supports the theory of a possible downstaging effect of preoperative chemotherapy’ [5], are very difficult to classify in the context of evidence-based medicine). The Innovation Indicators can allow for visualisation of the paucity of attempts to ‘take additional ground’ in the matrix and thus drive forward our understanding of the full spectrum of treatment effects.

In addition to more systematic efforts to increase understanding of single drugs after they enter the market, we also need to think about incentives to combine treatments in order to ‘move up’ in the matrix. Currently, there appears to be a lack of systematic investigations in this realm in spite of the clear success that combination treatments have achieved in some tumours. We are currently asking stakeholders to provide their own values for the different squares of the matrix (right side of Figure 1) to uncover the variety of value models that may exist and establish a set of defaults for the first data release on 10 common tumour types.

### Policy discussion II—biomarkers

Curing late-stage cancer (square ‘A’ in the matrix) is clearly a desirable goal. Developing new treatments that aim at this square is one way to reach it, but there are other ways that aim at optimising our understanding and the use of existing treatments. Disease-tracking biomarkers, for example, could tell us which patients in the current heterogeneous treatment populations have residual tumours or micro-metastases that need continuous aggressive treatment, and which are truly disease-free.

The adjuvant treatments in current use in the ‘J’ square (Figure 2) obviously do not increase survival by treating those patients who are ‘cured’ by surgeons. They benefit those individuals who harbour residual disease, but efforts to identify these (e.g. by circulating tumour cells) have not yet led to any demonstrable differences in treatment approaches that caused significantly different outcomes in clinical trials. This has been frustrating, and Hayes *et al* [6] recently concluded, ‘The marketplace has recognised the value of advances in cancer care that have resulted from the discovery and development of molecularly targeted therapies but not the value of robust new tumour-biomarker tests to guide patient management’.

Not only could the cancer policy field learn a lot from the ability to track treatment response based on molecular phenotypes, but also save considerable resources by no longer (over-) treating those who do not require it. The Innovation Indicators can be used to visualise these unmet needs and serve as a tool in discussions regarding possible missing incentives to accelerate biomarker discovery efforts and other areas of research that can move existing pieces into higher levels of the matrix.
